# On-Site Identification of Corrosion Products and Evaluation of the Conservation Status of Copper Alloy Artworks Using a Portable Raman Spectrometer

**DOI:** 10.3390/ma18050924

**Published:** 2025-02-20

**Authors:** Heehong Kwon, Leeyun Kim

**Affiliations:** Department of Conservation and Art Bank, National Museum of Modern and Contemporary Art, Korea, Cheongju-si 28501, Republic of Korea; iyeon1996@kore.kr

**Keywords:** portable Raman spectroscopy, copper alloys, corrosion products, outdoor corrosion, patina, non-destructive investigation, in situ application

## Abstract

Copper alloys form various corrosion products such as sulfides and chlorides. Chlorides can cause severe structural damage in ‘bronze disease’, making the early identification of corrosion products and conservation treatment important tasks. In this study, standard spectra were established for nine minerals of corrosion products using a portable Raman spectrometer, and their identification was verified by comparing them with benchtop micro-Raman spectra. The main characteristic bands were detected for most corrosion products, and the in situ applicability of the portable Raman spectrometer was demonstrated. However, for some samples, the signal-to-noise ratio was low, while the main characteristic peaks were still identifiable. In particular, dicopper chloride trihydroxides (such as atacamite and clinoatacamite) were clearly distinguished as corrosion products whose early identification is crucial. After the on-site analysis of copper alloy artworks exposed to outdoor environments for over 30 years, corrosion products such as malachite, brochantite, and moolooite were detected, indicating that portable Raman spectrometers are an effective tool for diagnosing conservation conditions. This study demonstrates that portable Raman spectrometers can be effectively used to identify corrosion products and assess the conservation state of copper alloy artworks and are expected to make significant contributions to future conservation and restoration efforts.

## 1. Introduction

Cultural heritage and artworks are significant assets that reflect the history and traditions of humanity, and the conservation of these artifacts is a primary concern for numerous conservators and researchers. The artworks composed of copper alloys are prone to various forms of corrosion, including oxidation, sulfidation, and chlorination, which are promoted by environmental factors such as oxygen, moisture, organic compounds, and atmospheric pollutants.

Previous studies have primarily classified the corrosion products of outdoor copper alloys into sulfides and chlorides, and the corrosion mechanisms of these compounds have been examined in detail [1,2,3,4,5,6,7,8,9,10,11,12,13]. In particular, sulfide patina forms a protective layer that imparts esthetic and historical values to copper alloy artworks; however, the excessive accumulation of sulfate (SO_4_) ions may induce their corrosion. Additionally, chloride patina can cause serious corrosion issues such as pitting corrosion and bronze disease, which should be detected early, and appropriate conservation treatments must be performed. Furthermore, dicopper chloride trihydroxide is formed rapidly within a short period, weakly adheres to the underlying layer, possesses a highly porous crystalline structure, and causes a volume expansion, which can induce external deformation and internal physical stress in copper alloy artworks and cause structural problems [12,14,15].

These studies provided important foundational data for a proper understanding of the corrosion mechanisms of copper alloys; however, because most of them were conducted in a laboratory, they exhibited limited practical on-site analysis capabilities. Traditional high-resolution analytical techniques [10,16,17,18,19] such as scanning electron microscopy (SEM)—energy-dispersive spectroscopy (EDS), X-ray diffraction (XRD), and micro-Raman spectroscopy, demonstrate high precision and reliability; however, these methods are expensive and require long analysis times, making immediate on-site analysis a difficult task.

Accordingly, the demand for facile on-site analysis techniques has been growing recently, and the development of portable analytical equipment is currently underway. In particular, portable Raman spectrometers can be used for rapid compositional on-site analysis with potential applications in the field of conservation science [13,20,21,22,23,24,25,26,27]. Raman spectroscopy is a non-destructive analytical method based on the molecular vibrations of substances, which is particularly effective for analyzing complex compounds. However, there is a lack of research studies on the accuracy and reliability of portable Raman spectrometers for complex metal corrosion products such as the corrosion products of copper alloys. In addition, they may have limited sensitivity and resolution compared to benchtop spectrometers, and are susceptible to changes in external conditions, which may cause background noise.

In this study, we investigate the potential of using portable Raman spectrometers to analyze various corrosion products of copper alloys (e.g., copper oxide, copper sulfide, copper chloride, and copper carbonate). This involves assessing the accuracy of the portable equipment and validating its identification capabilities through comparative analyses with benchtop micro-Raman spectrometers. Additionally, auxiliary analytical methods such as SEM-EDS are considered to enhance the reliability of portable Raman spectrometers for corrosion analysis.

The portable Raman spectroscopy approach allows for practical on-site examinations of copper alloy artworks, some of which have been exposed to outdoor environments for over 30 years. The goal is to facilitate early detection of corrosion and effectively assess the conservation status of these artworks.

The outcomes of this research provide valuable insights into the practical application of portable Raman spectrometers. These findings support ongoing efforts to efficiently monitor corrosion processes and manage the conservation of cultural heritage artifacts, thereby aiding in their preservation and enhancing the usability of portable on-site analysis tools.

The specific aims of this research are as follows:

1. Validate the feasibility of a technique for rapid and non-destructive on-site identification of various corrosion products on copper alloy artworks using a portable Raman spectrometer.
2. Compare the results of the portable Raman spectrometer analysis with those from a benchtop Raman spectrometer analysis to evaluate the accuracy and reliability of the portable equipment.
3. Conduct on-site analysis of actual copper alloy artworks exposed to long-term outdoor environments to verify the practicality of identifying corrosion products and assessing their preservation status using a portable Raman spectrometer.

## 2. Methods

### 2.1. Constructing Standard Spectra with a Portable Raman Spectrometer

#### 2.1.1. Standard Compounds

**Figure 1:**
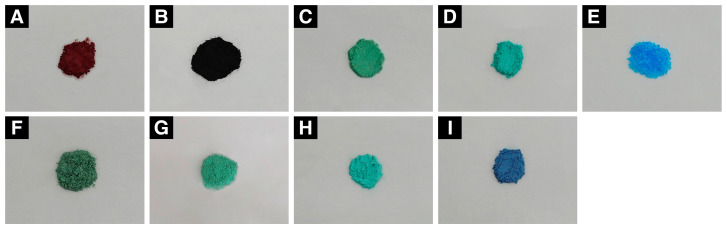
Photographs of the standard reference corrosion products of the copper alloy: (**A**) cuprite, (**B**) tenorite, (**C**) antlerite, (**D**) brochantite, (**E**) chalcanthite, (**F**) atacamite, (**G**) clinoatacamite, (**H**) malachite, and (**I**) azurite.

Standard spectra were established for various commercially available copper minerals and powders. Selected standard samples included nine compounds: copper oxides (cuprite and tenorite), copper sulfides (antlerite, brochantite, and chalcanthite), copper chlorides (atacamite and clinoatacamite), and copper carbonates (malachite and azurite) (Figure 1, Table 1). Standard corrosion products manufactured by Sigma-Aldrich^®^ (St. Louis, MO, USA) and Kremer Pigment Inc. (New York, NY, USA) are recognized as chemical compositions and analytically pure reagents and are used in corrosion identification research on cultural heritage [28,29,30].

Below are the chemical equations of selected standard minerals [17,31,32]:

2Cu^+^ + 2OH^−^ → Cu_2_O + H_2_O (cuprite) (1)

2Cu + 1/2O_2_ → 2CuO (tenorite) (2)

6Cu_2_O + 4SO_2_ + 5O_2_ + 8H_2_O → 4Cu_3_(SO_4_)(OH)_4_ (antlerite) (3)

4Cu_2_O + 2SO_2_ + 3O_2_ + 6H_2_O → 2Cu_4_(SO_4_)(OH)_6_ (brochantite) (4)

CuO + H_2_SO_4_ + 4H_2_O → CuSO_4_·5H_2_O (chalcanthite) (5)

4CuCl + 4H_2_O + O_2_ → 2Cu_2_Cl(OH)_3_ + 2HCl (atacamite, clinoatacamite) (6)

2Cu_2_O + 2CO_2_ + O_2_ + 2H_2_O → 2Cu_2_(CO_3_)(OH)_2_ (malachite) (7)

6Cu_2_O + 8CO_2_ + 3O_2_ + 4H_2_O → 4Cu_3_(CO_3_)_2_(OH)_2_ (azurite) (8)

#### 2.1.2. Analysis Techniques

A scanning electron microscope (JSM-6610LV, JEOL, Tokyo, Japan) was used to observe the microstructures of corrosion products. The SEM images were analyzed in the secondary electron image mode at a voltage of 10 kV and working distance of 13 mm. In addition, a colorimetric analysis was conducted using a spectrophotocolorimeter (CR-700d, Minolta, Tokyo, Japan) in the specular-component-excluded mode at a measurement diameter of 3 mm.

A benchtop Raman spectrometer (LabRam HR Evolution, Horiba Jobin-Yvon, Kyoto, Japan) was used to evaluate the applicability of the portable Raman spectrometer. The corrosion products’ Raman spectra were obtained with the 532 nm wavelength diode laser. The laser power was 14 mW, and the spectral resolution was about 2 cm^−1^. The diffraction grating was 600 g/mm, neutral density filters (NDFs) were D1.3, D1.5 and D3, objective magnification was 100×. The scan range was 100–4000 cm^−1^, and data were examined only in the range from 100 to 2000 cm^−1^, in which characteristic peaks of the corrosion products depended on the corrosion type.

A portable Raman spectrometer (I-Raman Pro, B&W Tek, Newark, NJ, USA) was operated using a 35 mW diode laser with a wavelength of 532 nm in a spectral range of 100–3457 cm^−1^ at a resolution of 8 cm^−1^. The scanning time for each Raman spectrum was set to 10 s, and to improve the signal-to-noise ratio, the results of 3–5 scans were combined for each experimental run. This was limited to allow immediate identification and applicability evaluation in field environments. Furthermore, spectral reproducibility was verified by performing analyses at multiple points (2–3 points) on the same sample [33]. The results obtained by the portable and benchtop Raman spectrometers were verified by comparing them with the standard data from the RRUFF™ Project and previous research findings.

The use of a 532 nm laser in the Raman spectrometer allows for efficient excitation of Raman scattering of many materials, including copper oxides and other copper corrosion products. The 532 nm laser wavelength is a compromise between shorter wavelength radiation (e.g., 488 nm), which is more likely to cause luminescence, and longer wavelength radiation (e.g., 785 and 1064 nm), which according to Rayleigh’s law (I~λ-4, where I is the intensity of Raman scattering and λ is the wavelength) for intensity leads to lower Raman scattering intensity. Raman spectrometers operating at wavelengths of 785 and 1064 nm are effective only under particular conditions and may be less efficient for the analysis of copper compounds. Importantly, it is challenging to detect peaks within specific wavelength ranges for the identification of chloride corrosion products [20]. Hence, a wavelength of 532 nm is more suitable for analyzing corrosion products.

### 2.2. Evaluation of the In Situ Applicability of a Portable Raman Spectrometer

#### 2.2.1. Research Objects

The standard spectrum obtained using a portable Raman spectrometer was used to evaluate the possibility of non-destructively identifying corrosion products formed on the surfaces of the actual outdoor bronze sculptures. In particular, three copper alloy artworks that have been exposed to urban–industrial environments for over 30 years were selected (Table 2). Around 500 bronze sculptures made in Republic of Korea or other countries since the 20th century were made of a quaternary alloy (Cu–Zn–Sn–Pb) [11,12,13,34,35,36,37,38,39,40].

#### 2.2.2. Analysis Techniques

The chemical compositions of the artworks were analyzed in situ using a portable X-ray fluorescence analyzer (DPO-2000, Olympus, Center Valley, PA, USA). The analysis conditions included a collimator size of 10 mm, voltage of 40 kV with an alumina filter for 10 s, and voltage 8 kV without a filter for 30 s. Portable Raman spectroscopy was performed under the same conditions as those utilized to construct the standard spectrum of the copper alloy. A portable Raman spectrometer with 785 nm laser wavelength excitation was used for the materials analysis (see SC3 in Table 2) in case of inability to detect the Raman peaks with portable spectrometer with 532 nm laser wavelength excitation. A portable Raman spectrometer (Inspector Raman, Deltanu, Daytona Beach, FL, USA) with a 60 mW diode laser was used in a spectral range of 200–2000 cm^−1^ at a resolution of 8 cm^−1^.

## 3. Results of Standard Compounds Analysis

### 3.1. Copper Oxides

**Figure 2:**
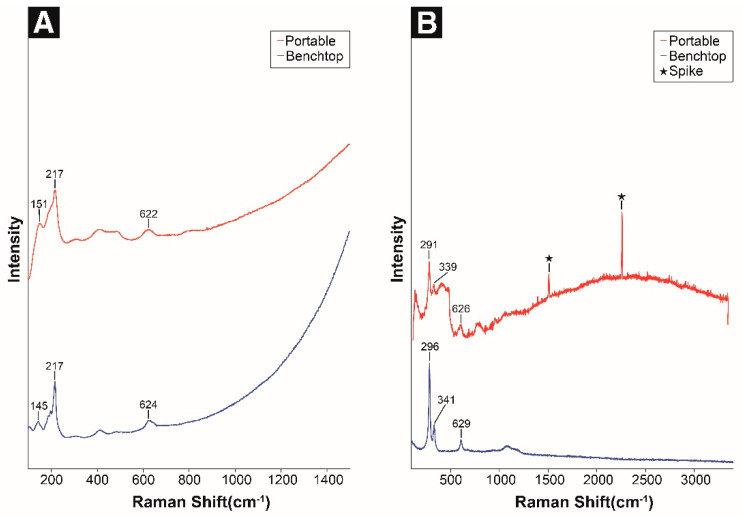
Raman spectra of standard copper oxides: (**A**) cuprite and (**B**) tenorite.

Cuprite (Cu_2_O) is the most common corrosion product of copper alloys exposed to the atmosphere, forming a reddish or reddish-brown layer on the metal surface. The optical properties of cuprite are less affected by the environment, and impurities such as hydrogen present in the patina layer on the bronze surface migrate into the cuprite layer and considerably affect its defects and semiconductor properties [7,11]. Using the portable Raman spectrometer, the characteristic peaks at 151 (O-Cu-O bend), 217 (Cu-O bond), and 622 cm^−1^ (Cu-O vibration) were identified [10,18,19], which closely matched the main peaks obtained by the benchtop micro-Raman spectroscoper, and the corresponding peak shapes were also similar (Figure 2A, Table 3). In particular, the 622 cm^−1^ peak features metal–oxygen place exchange and is also referred to as a native passive layer of copper (defective Cu_2_O) [18].

Tenorite (CuO) is formed when copper is slowly heated to 400–600 °C in the atmosphere. Generally, the presence of tenorite indicates that archeological artifacts were heated due to a fire or cooking before or during the burial process [41]. Using the portable Raman spectrometer, the spectral characteristics of tenorite were confirmed, although a relatively strong noise signal was detected as compared with the spectrum obtained by the benchtop micro-Raman spectrometer. Notably, the Raman bands at 291, 339, and 626 cm^−1^ were observed (Figure 2B, Table 3). However, compared with other standard corrosion product samples, the signal-to-noise ratio was low, and the noise level was relatively high. These differences can be attributed to the difference in Raman scattering cross-section of the materials as well as their amounts. It is also influenced by various factors such as analytical conditions and equipment characteristics [20,26].

**Table 3:** Raman spectral peak positions of copper oxides with reference values. The intensities measured for the corrosion products are reported as follows: (v.s) very strong, (s) strong, (m) medium, (w) weak.

Cuprite (cm^−1^)			Tenorite (cm^−1^)		
Portable	Benchtop	Reference [42]	Portable	Benchtop	Reference [43]
622	624	625 (w)	626	629	629 (s)
			339	341	341 (m)
			291	296	296 (v.s)
217	217	217 (v.s)			
151	145	145 (m)			
		108 (m)			

### 3.2. Copper Sulfides

**Figure 3:**
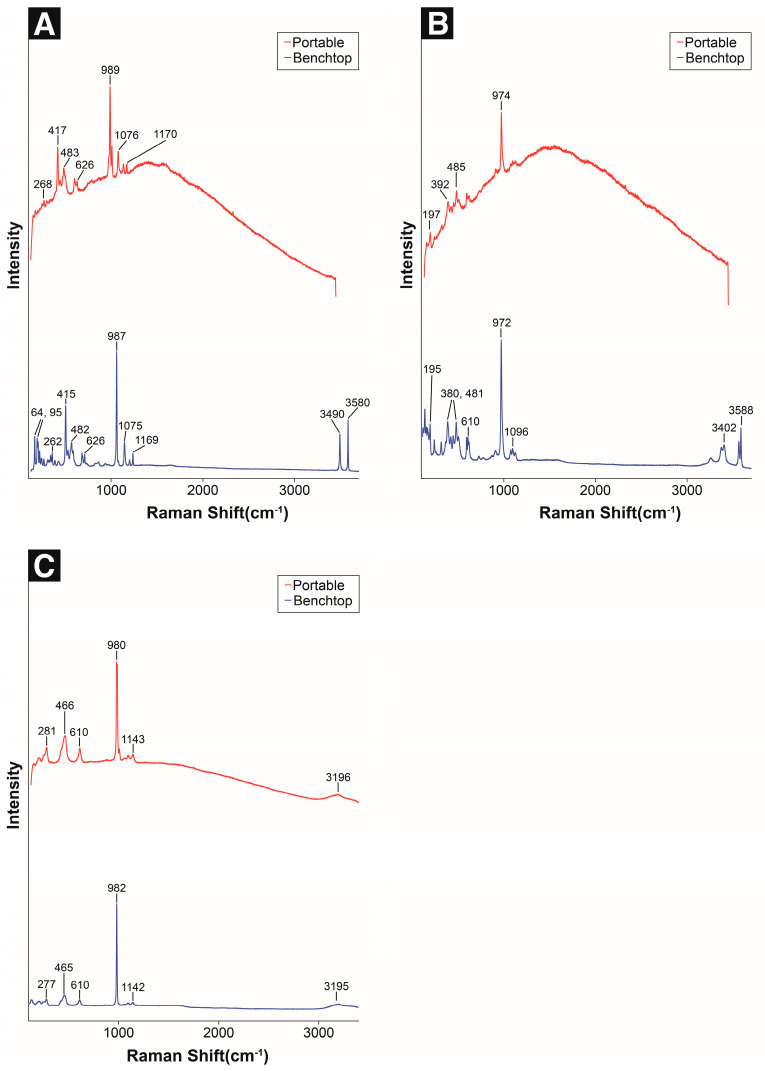
Raman spectra of standard copper sulfides: (**A**) antlerite, (**B**) brochantite, and (**C**) chalcanthite.

Antlerite (Cu_3_SO_4_(OH)_4_) is a corrosion product indicating a low-pH environment. Since the mid-20th century, it has been frequently found in the corrosion layers of copper alloys as the pH of rainwater has become more acidic [41]. Using the portable Raman spectrometer, the Raman bands at 417, 483, 989, and 1076 cm^−1^ were identified, which closely matched the main peaks obtained by the benchtop micro-Raman spectrometer, and the corresponding peak intensity ratio was also similar (Figure 3A, Table 4).

Brochantite (Cu_4_SO_4_(OH)_6_) is known as the most stable and common corrosion product on bronze sculptures exposed to the atmosphere [2,11,41,44], which remains stable under various chemical conditions [2,45]. By utilizing portable Raman spectroscopy, three main bands (392, 485, and 974 cm^−1^) were identified, and their peak intensity ratios were similar to those of the main peaks obtained via benchtop micro-Raman spectroscopy (Figure 3B, Table 4). Although some differences were observed in the range of 130–600 cm^−1^, the characteristic peaks of brochantite were identified.

Chalcanthite (CuSO_4_·5H_2_O) is a secondary mineral formed in the oxidation zones of copper sulfide deposits and volcanic activity areas, which exists in radial fibrous structures or stalactite forms [44,45,46]. Using portable Raman spectroscopy, the Raman bands at 281, 466, 610, 987, and 1145 cm^−1^ were identified. This result was almost identical to the spectrum obtained via benchtop micro-Raman spectroscopy (Figure 3C, Table 4).

Antlerite, brochantite, and chalcanthite exhibit similar main peaks 973, 982, and 994 cm^−1^ (SO_4_^2−^ symmetric stretch) [19,20,21,22], which are difficult to identify at a low signal-to-noise ratio. However, chalcanthite has different peak frequencies in the 380–485 and 600–630 cm^−1^ ranges as compared with those of antlerite and brochantite, and a very weak band is observed in the 3190–3195 cm^−1^ region. Therefore, it is possible to distinguish between the two corrosion products when the noise level is relatively low.

However, antlerite and brochantite were difficult to identify owing to the limited spectral range and low resolution. In particular, the difficulty of specific peak detection in the range of 130–600 cm^−1^ is attributed to the instrument resolution. Possible solutions include the utilization of a high-resolution benchtop micro-Raman spectrometer, peak overlap minimization through principal component analysis, sample pretreatment, and the adjustment of the analytical conditions [47,48].

When the signal-to-noise ratio decreases, similar spectral patterns overlap, making their identification a difficult task. This issue can become more pronounced depending on the environmental conditions and presence of contaminants. For example, the roughness of the sample surface or presence of oxide layers can decrease the measurement accuracy. To address this issue, it is necessary to improve signal quality through stricter environmental control and appropriate sample pretreatment [47].

These two corrosion products have similar chemical compositions; however, their formation mechanisms and environmental factors are also important. The formation of antlerite and brochantite occurs under different pH conditions with antlerite primarily produced in low-pH environments. As a result, the formation processes and physicochemical properties of the two substances differ significantly, making their identification a more complex task.

In previous studies, the overall band patterns of the two corrosion products were similar, and methods for distinguishing antlerite and brochantite by monitoring certain bands (400–420 and 1000–1100 cm^−1^) were proposed [13,21]. Additionally, several attempts were made to differentiate between these minerals by controlling the measurement environment (e.g., at temperatures higher than room temperature) [49]. However, antlerite (pH = 2.8–3.5) is formed in more contaminated environments than brochantite (pH = 3.5–6.5); therefore, special care is required to prevent damage to copper alloys. This suggests that the patina layer on the copper alloy artworks exposed to the atmosphere can be dissolved, causing streaks and surface damage to the pieces [41,50].

**Figure 4:**
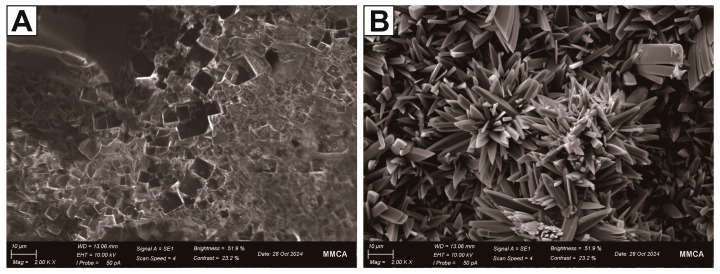
Scanning electron microscopy of standard copper corrosion (SEM) images of copper sulfide crystals: (**A**) antlerite and (**B**) brochantite.

Therefore, to preserve patinas with esthetic and historical values, the accurate identification of corrosion products is essential, and auxiliary analytical methods such as SEM–EDS and XRD should be considered as the intermediate analytical approaches. In this study, a trace amount (0.1 g) of each sample was observed via SEM. According to Figure 4, antlerite exhibits polygonal and orthorhombic crystals with sizes ranging from approximately 10 to 40 µm, whereas brochantite forms small rod-shaped crystals with sizes of 10–20 µm, which aggregate into prismatic or massive clusters constituting large flower-shaped crystal assemblies.

**Table 4:** Raman spectral peak positions of copper sulfides with reference values. The intensities measured for the corrosion products are reported as follows: (v.s) very strong, (s) strong, (m) medium, (w) weak, and (v.w) very weak.

Antlerite (cm^−1^)			Brochantite (cm^−1^)			Chalcanthite (cm^−1^)		
Portable	Benchtop	Reference [51]	Portable	Benchtop	Reference [52]	Portable	Benchtop	Reference [53]
	3580	3583 (m)		3588	3590 (s)			
				3565	3569 (w)			
	3490	3492 (m)						
				3402	3405 (s)			
				3372	3380 (v.w)			
				3260	3263 (v.w)			
						3196	3195	3194 (m)
1170	1169	1178 (m)						
1135	1133	1141 (w)				1143	1142	1143 (v.w)
			1126	1126	1106 (w)			
			1094	1096		1094	1096	1094 (v.w)
1076	1075	1083 (m)						
			1074	1076	1077 (w)			
989	987	994 (v.s)						
						980	982	982 (v.s)
			974	972	973 (v.s)			
			911	901	909 (w)			
	862	867 (v.w)						
				779	774 (v.w)			
				727	729 (v.w)			
626	626		622	620				
603	599	609 (m)		610	610 (s)	610	610	610 (w)
			597	596				
				505				
483	482	482 (m)	485	481	483 (s)			
						466	465	464 (m)
				449				
		424 (s)		422	424 (v.w)			
417	415							
			392	380	387 (s)			
			322	318	319 (m)			
	340	342 (w)						
						281	277	275 (w)
268	262	273 (w)						
		256 (w)						
				242	242 (m)			
		227 (w)						
			197	195				
						207	199	
173	172							
			157		150 (m)			
						149		148 (w)
		133 (w)		138				
							126	
	95							
	64							

### 3.3. Copper Chlorides

**Figure 5:**
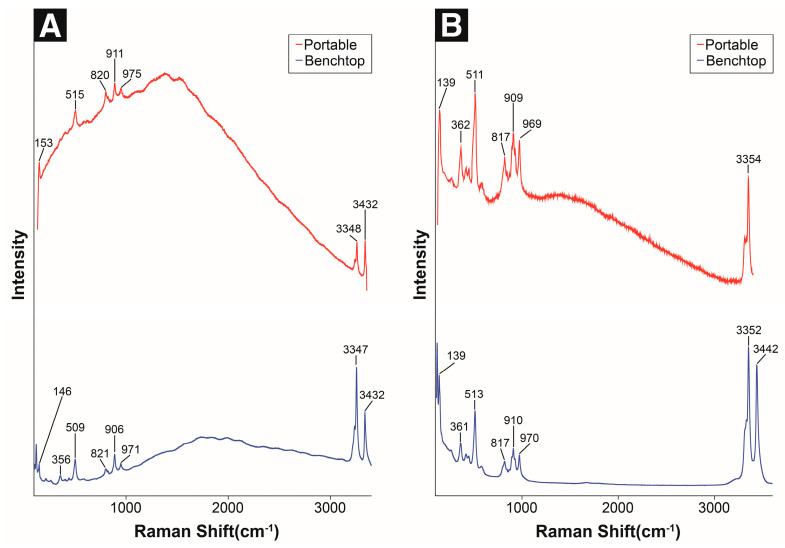
Raman spectra of standard copper chlorides: (**A**) atacamite and (**B**) clinoatacamite.

Atacamite is the most common corrosion product among the dicopper chloride trihydroxide (Cu_2_Cl(OH)_3_) polymorphs. It is more frequently found in outdoor bronze sculptures than paratacamite, and both compounds are sometimes detected simultaneously under specific conditions. Atacamite is difficult to identify by XRD when it is mixed with other corrosion products [2,54,55]. Using portable Raman spectroscopy, the Raman bands at 153, 515, 820, 911, 975, 3348, and 3432 cm^−1^ were identified, which closely matched the main peaks obtained by the benchtop micro-Raman instrument, and the corresponding peak shapes were also similar (Figure 5A, Table 5).

Clinoatacamite is thermodynamically more stable than other dicopper chloride trihydroxide polymorphs (atacamite, paratacamite, and botallackite) and represents the final corrosion product. In previous studies of marine atmospheric corrosion environments, atacamite has always been undetected, whereas clinoatacamite has always been identified and is formed at high chloride concentrations [16]. Using portable Raman spectroscopy, the Raman bands at 139, 262, 511, 817, 909, 969, and 3354 cm^−1^ were identified. These bands exhibit a similar trend to the main peaks observed via micro-Raman spectroscopy (Figure 5B, Table 5).

After analyzing dicopper chloride trihydroxide polymorphs, atacamite and clinoatacamite produced four strong Raman bands in specific regions (140–150, 510, 810–980, and 3300–3500 cm^−1^) with slight differences in some areas. These correspond to the characteristic O–Cu–O band (140–150 cm^−1^), CuO band (510 cm^−1^), and OH deformation (810–980 cm^−1^), respectively [20,56,57].

Unfortunately, a standard paratacamite sample could not be used in this study; however, the paratacamite Raman spectrum is different from those of atacamite and clinoatacamite. Atacamite and clinoatacamite exhibit three strong characteristic peaks in the 810–980 cm^−1^ range due to OH deformation, whereas paratacamite produces a single strong band near 940 cm^−1^, distinguishing it from the other two compounds [21]. This indicates that paratacamite is a separate phase rather than a dimorphous form of atacamite or clinoatacamite [16,21]. This distinction can be attributed to the unique structural and chemical properties of paratacamite.

Additionally, unlike the unidentified sulfide corrosion products (such as antlerite and brochantite), chloride corrosion products including dicopper chloride trihydroxide isomers (atacamite and clinoatacamite) were commonly detected in the 3300–3450 cm^−1^ range owing to the limited wavenumber range of portable Raman spectroscopy. This suggests that portable Raman spectrometers can be used as important analytical tools to identify the corrosion types of chlorides and sulfides and quickly diagnose their conservation states in outdoor settings.

**Table 5:** Raman spectral peak positions of copper chlorides with reference values. The intensities measured for the corrosion products are reported given as follows: (v.s) very strong, (s) strong, (m) medium, (w) weak, and (v.w) very weak.

Atacamite (cm^−1^)			Clinoatacamite (cm^−1^)		
Portable	Benchtop	Reference [58]	Portable	Benchtop	Reference [59]
				3442	3444 (v.s)
3432	3432	3428 (v.s)			
			3354	3352	3355 (v.s)
3348	3347	3342 (v.s)			
			3310	3305	3307 (s)
975	971	968 (m)	969	970	969 (m)
			929	930	929 (m)
911	906	906 (m)	909	910	
				891	895 (m)
820	821	814 (m)	817	817	
				801	800 (w)
		590 (v.w)			
			579	575	577 (w)
515	509	509 (m)	511	513	510 (m)
	446	446 (v.w)	448	447	446 (w)
			420	415	421 (w)
410	410	410 (v.w)			
			362	361	366 (m)
356	356	354 (w)			
	266	262 (v.w)	262	264	262 (v.w)
239		244 (v.w)			
		228 (v.w)			
	217				
					166 (v.w)
153	146	141 (m)	139	139	140 (m)
	115			115	

### 3.4. Copper Carbonates

Basic copper carbonate appears as a natural patina on bronze sculptures exposed to atmospheric corrosion, which is formed through the reaction between the patina surface and CO_2_ in the atmosphere or HCO_3_^−^ ions dissolved in the condensed moisture. However, because the concentrations of CO_2_ and HCO_3_^−^ species in the atmosphere are low, basic copper carbonate is rarely detected [60]. Malachite and azurite are secondary minerals formed in the weathering zone of copper ore deposits. They are generally associated with each other and frequently found alongside other copper minerals [61].

**Figure 6:**
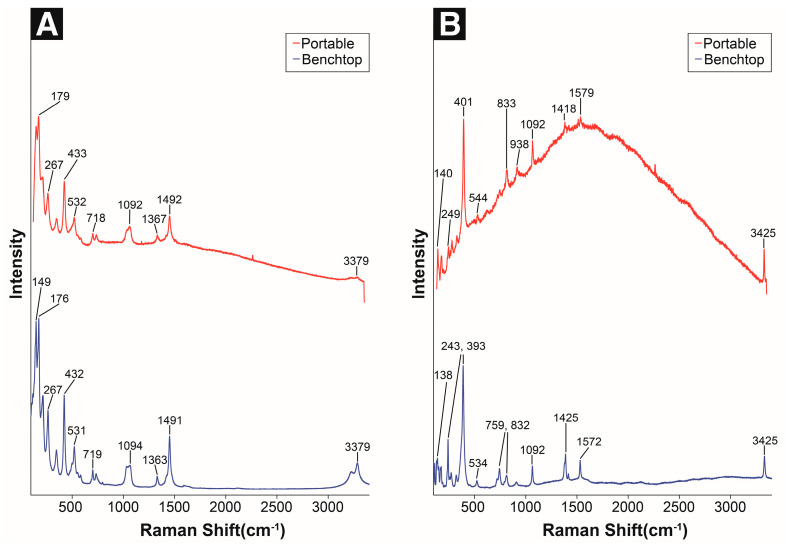
Raman spectra of standard copper carbonates: (**A**) malachite and (**B**) azurite.

Malachite (Cu_2_(CO_3_)(OH)_2_) is primarily identified in archeological burial artifacts; however, it can also be formed in the presence of chlorides in clean and humid air. Furthermore, malachite may also be produced through the reaction of CO_2_ and cuprite in the atmosphere. It is found in some outdoor bronze sculptures but rarely observed because it is replaced by copper hydroxysulfate owing to the presence of sulfur dioxide in the atmosphere [3,4,6,62]. Using the portable Raman spectrometer, the Raman shifts of 150, 179, 433, 718, 1092, 1492, 3379 cm^−1^ were identified, which closely matched the main peaks obtained via benchtop micro-Raman spectroscopy, and the corresponding peak shapes were similar (Figure 6A, Table 6).

The band at 150–433 cm^−1^ is predicted to be generated by the extrinsic vibrations between the [CO_3_]^2−^/[OH]^−^ units associated with Cu^2+^ (basically ~300–600 cm^−1^) and the vibrational band of the [CO_3_]^2−^ unit (below ~300 cm^−1^). Raman modes occurring in the frequency range of 700–1600 cm^−1^ are due to the [CO_3_]^2−^ internal modes. The Raman shifts of 3379 cm^−1^ represents the O–H stretching region [63,64].

Azurite (Cu_3_(OH)_2_(CO_3_)_2_) is also unstable in the atmosphere and tends to transform into malachite when CO_2_ levels are low and H_2_O levels are high [60,62]. Similarly to malachite, azurite can appear as a dense thin layer in copper alloy artifacts, but is more frequently observed in the form of fine blue crystal aggregates scattered between malachite crystals [65]. Using the portable Raman spectrometer, the Raman bands at 140, 401, 833 (Out-of-phase and in-phase bending modes of CO_3_^2−^), 1092 (CO symmetric stretching vibration), 1579 (CO_3_^2−^ antisymmetric stretching vibration), and 3425 (O-H stretching) cm^−1^ were identified [66]. These bands exhibit a similar trend to the main peaks observed via benchtop micro-Raman spectroscopy (Figure 6B, Table 6).

**Table 6:** Raman spectral peak positions of copper carbonates with references. The intensities measured for the corrosion products are reported as follows: (v.s) very strong, (s) strong, (m) medium, (w) weak, and (v.w) very weak.

Malachite (cm^−1^)			Azurite (cm^−1^)		
Portable	Benchtop	Reference [67]	Portable	Benchtop	Reference [68]
			3425	3425	3428 (s)
3379	3379	3379 (s)			
3318	3314	3310 (m)			
					1651 (v.w)
			1579	1576	1582 (w)
1492	1491	1489 (v.s)			
			1457	1458	1462 (v.w)
			1421	1428	1430 (m)
1367	1363	1364 (w)			
1092	1094	1092 (m)	1092	1092	1096 (m)
1062	1056	1060 (m)			
			938	933	938 (v.w)
			833	834	837 (m)
			762	759	761 (m)
753	749	747 (w)			
718	719	719 (w)			
595	597	596 (w)			
532	531	532 (s)	544	534	542 (w)
433	432	432 (v.s)			
			401	398	401 (v.s)
357	353	352 (w)			
			336	324	
267	267	266 (v.s)	285	276	276 (w)
			249	246	247 (m)
213	215	216 (w)			
179	176	175 (v.s)	176	173	171 (m)
150	149	152 (s)			
			140	138	135 (m)

## 4. In Situ Applicability of Portable Raman Spectrometers to Copper Alloy Artworks

**Figure 7:**
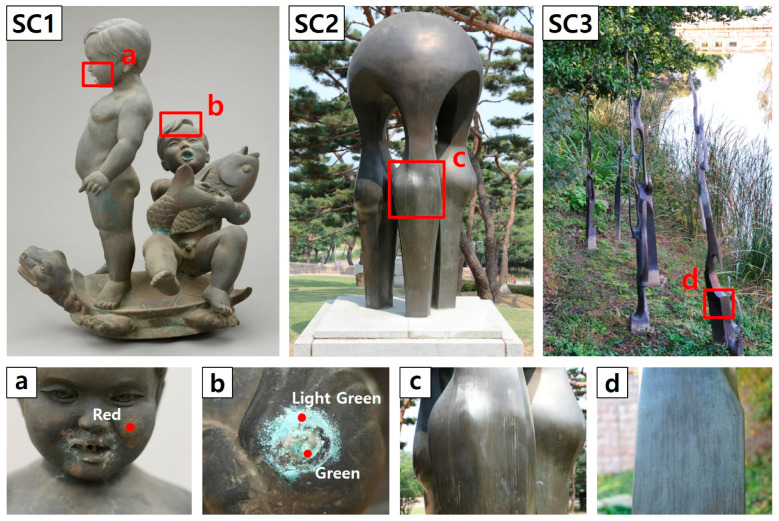
Artworks studied via portable Raman spectroscopy: (**a**,**b**) SC1, (**c**) SC2 and (**d**) SC3.

In this study, nine types of corrosion products (including cupric oxide, cuprous sulfide, cuprous chloride, and cupric carbonate) commonly found in copper alloy artworks exposed to outdoor environments for extended periods were analyzed using a portable Raman spectrometer. In this analysis, the corrosion products of three copper alloy artworks (Figure 7) exposed to the outdoors for over 30 years were identified, and the possible presence of each corrosion product was examined.

**Figure 8:**
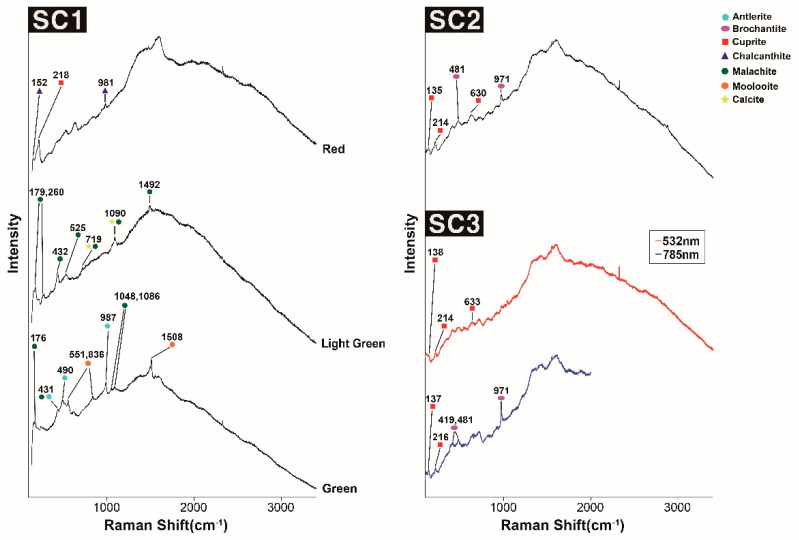
Portable Raman spectroscopy data: SC1, SC2, and SC3.

SC1 is a Cu–Zn–Sn–Pb quaternary bronze sculpture, which was exposed to outdoor environments for an extended period (SC1 in Figure 7, Table 2). The sculpture has a hole, through which water flows, suggesting that it was used as a fountain in the past. In the corrosion products denoted in red in Figure 7a(SC1), the Raman bands at 152, 218, 525, 981, and 1472 cm^−1^ were identified, confirming the presence of cuprite (152 and 218 cm^−1^) and chalcanthite (152 and 981 cm^−1^) corrosion products. Furthermore, X-ray fluorescence analysis results showed the presence of calcium (Table 2), which suggests the formation of calcium oxalate (CaC_2_O_4_·H_2_O) [17]. According to Figure 7b(SC1), the locally formed corrosion products were visually distinguished as bright green and green areas. For the bright green corrosion products, the Raman bands corresponding to malachite (179, 260, 432, 525, 719, 1090 and 1492 cm^−1^) and calcite (281, 719, and 1090 cm^−1^) were identified (Figure 8(SC1), Table 4). Among the green corrosion products, malachite (176, 431, 1048 and 1086 cm^−1^), antlerite (490, 987 and 1086 cm^−1^), and moolooite (551, 836 and 1508 cm^−1^) were detected [62,69,70], (Figure 8(SC1), Table 7). However, the Raman spectra obtained in situ indicate that the presence of more than two corrosion products overlapping each other makes the distinction between different phases a challenging task, suggesting that additional analytical methods and considerable experience are required for their detection.

The presence of malachite, calcite, moolooite, and calcium oxalate indicates a prolonged exposure to a water-rich environment (such as a fountain), suggesting the possible presence of calcareous substances within the fountain and biological activity of fungi such as lichens. This indicates that SC1 made in the 1970s contains patina (antlerite and chalcanthite) as well as corrosion products in the carbonate series (such as malachite). Hence, the bronze sculpture was exposed for a long period in an outdoor environment with a fountain.

SC2 and SC3 are Cu–Zn–Sn–Pb quaternary bronzes that have been exposed to the outdoor environment for over 30 years (SC2 and SC3 in Figure 7, Table 2). In the case of SC3, the measurement results obtained using 785 nm and 532 nm lasers exhibited different peak intensities and shapes owing to the signal-to-noise ratio variations. The Raman shifts of cuprite (135–140, 214–216, and 630–640 cm^−1^) and brochantite (481 and 971 cm^−1^, SO_4_^2−^ symmetric stretching) were detected for both the SC2 and SC3 sculptures. The red spectrum of the SC3 portion of Figure 8 is thought to be cuprite, but its attribution is uncertain due to interference artifacts and requires further analysis. These results suggest that although the patina colors and surface conditions of the two sculptures are different, they were exposed to similar corrosion environments because of the similar alloy compositions and their proximity (within a radius of 300 m). The detection of cuprite in SC2 and SC3 suggests the presence of cupric oxide in the blue–green patina, while the detection of brochantite indicates a prolonged exposure to sulfur dioxide (SO_2_) pollutant in the urban-industrial environment of this area. However, the influence of other corrosion factors cannot be ruled out, which necessitates a further analysis in this area.

In conclusion, while SC1 generated various corrosion products owing to the continuous water contact, presence of calcite, and biological activity, SC2 and SC3 primarily formed brochantite and cuprite due to the SO_2_ exposure in a relatively dry environment. The location of each artifact, its past usage history, and the physical characteristics of its surface caused different corrosion environments, resulting in various corrosion products as well as patina colors and surface conditions.

## 5. Discussion

This study assessed the ability of a portable Raman spectrometer to rapidly and non-destructively identify the primary corrosion products of copper alloy artworks. The obtained results revealed that the performance of the portable Raman spectrometer was comparable to that of the benchtop micro-Raman spectrometer and successfully identified the Raman characteristic bands of the primary corrosion products. It was found that portable Raman spectrometers were highly applicable in the field and could serve as an effective instrument for the conservation and management of copper alloy artworks.

The portable Raman spectrometer demonstrated significant reliability in analyzing various corrosion products, including cupric oxide, cuprous oxide, cupric chloride, and cupric carbonate. The Raman bands of the chloride corrosion products, specifically atacamite and clinoatacamite, were distinctly identified in the field, illustrating the high application potential of the portable Raman spectrometer as a rapid and efficient diagnostic instrument in conservation science. This has considerable implications for the early identification of corrosion products that may lead to structural damage, such as the “bronze disease”.

A reduction in the signal-to-noise ratio was observed for certain corrosion products. For tenorite, the reliability of the Raman spectrum decreased owing to the significant noise level; however, the principal characteristic peaks remained discernible. This highlights the constraints of portable Raman spectrometers, necessitating future research studies aimed at optimizing analytical conditions and enhancing data processing techniques to improve the signal-to-noise ratio.

Furthermore, in the case of chalcopyrite, certain spectral discrepancies were detected during the identification of antlerite and brochantite. This indicates that Raman spectroscopy may exhibit a reduced accuracy in distinguishing corrosion products with similar spectral patterns; thus, a multi-analytical approach employing supplementary methods such as SEM–EDS may be employed to mitigate this issue.

In the analysis of copper alloy artworks, malachite, brochantite, and moolooite were identified as the primary corrosion products, and the portable Raman spectrometer demonstrated high efficacy in evaluating the corrosion condition of the artworks.

In conclusion, this study has demonstrated that portable Raman spectrometers are effective tools for identifying corrosion products in copper alloy artworks and suggests their potential utilization in conservation and diagnostic applications. Future research works should enhance the equipment performance and improve the analytical reliability across diverse environments, thereby contributing to the advancement of art conservation technology. Such works will help create innovative methodologies in conservation science and produce essential foundational data for developing effective conservation strategies in the future.

## 6. Conclusions

This study assessed the ability of a portable Raman spectrometer to rapidly and non-destructively identify the primary corrosion products of copper alloy artworks, and the results are as follows.

1. Identification of corrosion products: The portable Raman spectrometer successfully identified most of the corrosion products, including cuprite, tenorite, antlerite, brochantite, chalcanthite, atacamite, clinoatacamite, malachite, azurite, etc. In particular, atacamite and clinoatacamite, which cause serious corrosion problems, were identified with high accuracy.
2. Difficulty in identifying specific corrosion products: Differentiating anthrite and brochantite was difficult due to spectral similarities, and tenorite’s high noise level hindered analysis. A multi-analysis method was used to address these issues.
3. Field applicability evaluation: As a result of field analysis, malachite, brochantite, and moolooite were confirmed, proving the field applicability of the portable Raman spectrometer.

## Figures and Tables

**Table 1:** Properties of the standard corrosion products of the copper alloy.

Compound Class	Corrosion Product Name	Chemical Formula	Purity (%)	Manufacturer/Origin
Copper oxides	cuprite	Cu_2_O	97	Sigma-Aldrich^®^
	tenorite	CuO	99.9	Sigma-Aldrich^®^
Copper sulfides	antlerite	Cu_3_SO_4_(OH)_4_	N/A	Kremer Pigments Inc.
	brochantite	Cu_4_SO_4_(OH)_6_	N/A	Bisbee, AZ, USA
	chalcanthite	CuSO_4_·5H_2_O	≥98	Sigma-Aldrich^®^
Copper chlorides	atacamite	Cu_2_Cl(OH)_3_	N/A	Kremer Pigments Inc.
	clinoatacamite	Cu_2_Cl(OH)_3_	N/A	Aubazine, France
Copper carbonates	malachite	Cu_2_CO_3_(OH)_2_	99.9	Kremer Pigments Inc.
	azurite	Cu_3_(OH)_2_(CO_3_)_2_	N/A	Kremer Pigments Inc.

**Table 2:** Chemical compositions and colors of the corrosion products formed on artwork surfaces, which were determined via portable Raman spectroscopy.

No.	Artist	Artwork	Production Year	Elemental Composition (wt.%)					Corrosion Product Color		
				Cu	Zn	Sn	Pb	Ca	L*	a*	b*
SC1	Kyongok Kim	Peace	1970s	83.8	14.2	1.0	0.5	0.5	Light green		
									66.91	−13.16	−0.47
									Green		
									56.42	−10.98	5.36
									Red		
									42.44	0.55	4.71
SC2	Bonggoo Kim	Autumn	1986	85.2	8.9	3.5	2.0	-	Dark green		
									36.63	0.25	4.77
SC3	Youngjung Kim	Bud	1986	84.5	8.5	3.4	3.0	-	Dark green		
									29.02	−0.77	2.53

**Table 7:** Raman spectral peak positions of the outdoor copper alloy artworks.

SC1 (cm^−1^)			SC2 (cm^−1^)	SC3 (cm^−1^)	
Red	Light Green	Green		532 nm	785 nm
		1508			
	1492				
	1090	1086			
		1048			
981		987	971		971
		836			
	719				
641		639	630	633	639
		551			
525	525				
		490	481		481
	432	431			
			417		419
	358				
	281				
	260				
218	218		214	214	216
			193		
	179	176			
152		155			
			135	138	137

## Data Availability

The original contributions presented in the study are included in the article, further inquiries can be directed to the corresponding author.

## Abbreviations

The following abbreviations are used in this manuscript:

EDS	Energy-dispersive spectroscopy
XRD	X-ray diffraction
SEM	Scanning electron microscopy
